# Identification of *SOX18* as a New Gene Predisposing to Congenital Heart Disease

**DOI:** 10.3390/diagnostics12081917

**Published:** 2022-08-08

**Authors:** Hong-Yu Shi, Meng-Shi Xie, Chen-Xi Yang, Ri-Tai Huang, Song Xue, Xing-Yuan Liu, Ying-Jia Xu, Yi-Qing Yang

**Affiliations:** 1 Department of Cardiology, Zhongshan Hospital Wusong Branch, Fudan University, Shanghai 200940, China; 2 Department of Cardiology, Shanghai Fifth People’s Hospital, Fudan University, Shanghai 200240, China; 3 Department of Cardiovascular Surgery, Renji Hospital, School of Medicine, Shanghai Jiao Tong University, Shanghai 200127, China; 4 Department of Pediatrics, Tongji Hospital, Tongji University School of Medicine, Shanghai 200065, China; 5 Department of Cardiovascular Research Laboratory, Shanghai Fifth People’s Hospital, Fudan University, Shanghai 200240, China; 6 Department of Central Laboratory, Shanghai Fifth People’s Hospital, Fudan University, Shanghai 200240, China

**Keywords:** cardiology, congenital heart disease, genetics, transcription factor, reporter gene assay

## Abstract

Congenital heart disease (CHD) is the most frequent kind of birth deformity in human beings and the leading cause of neonatal mortality worldwide. Although genetic etiologies encompassing aneuploidy, copy number variations, and mutations in over 100 genes have been uncovered to be involved in the pathogenesis of CHD, the genetic components predisposing to CHD in most cases remain unclear. We recruited a family with CHD from the Chinese Han population in the present investigation. Through whole-exome sequencing analysis of selected family members, a new *SOX18* variation, namely NM_018419.3:c.349A>T; p.(Lys117*), was identified and confirmed to co-segregate with the CHD phenotype in the entire family by Sanger sequencing analysis. The heterozygous variant was absent from the 384 healthy volunteers enlisted as control individuals. Functional exploration via luciferase reporter analysis in cultivated HeLa cells revealed that Lys117*-mutant SOX18 lost transactivation on its target genes NR2F2 and GATA4, two genes responsible for CHD. Moreover, the genetic variation terminated the synergistic activation between SOX18 and NKX2.5, another gene accountable for CHD. The findings strongly indicate *SOX18* as a novel gene contributing to CHD, which helps address challenges in the clinical genetic diagnosis and prenatal prophylaxis of CHD.

## 1. Introduction

Congenital heart disease (CHD) represents the most frequent kind of human birth malformation, occurring in ~1% of all live newborns and up to 10% of stillbirths globally [1,2]. Notably, if mild cardiovascular structural anomalies are included, including bicuspid aortic valve as the most frequent cardiovascular developmental aberration with an estimated incidence of 1 to 2 per 100 of the general population, the total prevalence of CHD in live-born infants may be as high as ~5% [3]. As a collective diagnosis for cardiovascular developmental deformities, CHD is anatomically categorized into >20 different clinical subtypes, encompassing pulmonary stenosis (PS), patent ductus arteriosus (PDA), atrial septal defect, and hypoplastic left heart [1,4,5,6]. Although some mild CHD can resolve spontaneously, severe CHD often leads to worse quality of life associated with health [7,8,9], reduced exercise tolerance [10,11,12], brain injury and neurodevelopmental anomaly [13,14,15,16], thromboembolism [17,18], infective endocarditis [19,20], pulmonary arterial hypertension [21,22,23], chronic kidney disease and acute kidney injury [24,25,26], impaired liver function [27], restrictive lung dysfunction [28], congestive heart failure [29,30,31,32,33], miscellaneous cardiac dysrhythmias [34,35,36,37,38,39], and cardiac demise [40,41,42,43,44]. Striking improvement has been achieved in pediatric cardiac surgical procedures and perioperative intensive care as well as transcatheter interventional treatment over recent decades, which dramatically alters the natural history of CHD, allowing ~95% of children suffering from CHD to survive into adulthood, hence generating an ever-increasing population of adults reaching fertile age; at present, adults already outstrip the number of children living with CHD [45,46,47,48]. Surprisingly, the prolonged lifespan of CHD survivors is associated with greater long-term health risk, including increased vulnerability to cerebrovascular infarction, renal dysfunction, chronic heart failure, cancer, supraventricular and life-threatening ventricular arrhythmias, and sudden cardiac death [49,50,51,52]. Therefore, CHD has brought about substantially increased morbidity and mortality and has conferred a heavy socioeconomic burden on humans, highlighting the urgent need of identifying the etiologies responsible for CHD [1].

Cardiac organogenesis undergoes a complicated biological process that is finely controlled by a complex network, mainly comprising transcriptional factors, cardiac structural proteins, epigenetic modifying factors, signaling molecules as well as microRNAs [47,53]. It has been reported that both non-genetic environmental pathogenic factors and heritable defective components can disturb this sophisticated process, resulting in CHD [2,4,47,53,54]. Although an environmental contribution to CHD is unclear, environmental risk factors may contribute to approximately 10% of CHD [2]. Well-recognized non-heritable risk factors for CHD include, but are not limited to, maternal pre-gestational mellitus diabetes, viral infections, and exposures to medications during pregnancy [2,53]. However, ever-increasing evidence demonstrates that inherited determinants confer a paramount impact on the occurrence of CHD [2,4,47]. In addition to aneuploidies as well as copy number variants (gains and losses), pathogenic variations in over 100 genes, encompassing GATA4, NKX2.5, SOX7, and SOX17, have been implicated with the pathogenesis of CHD [2,4,47,55,56,57,58,59,60,61,62,63,64,65,66,67,68,69,70,71,72,73,74,75,76,77,78,79,80]. Nevertheless, in up to 55% of patients, the genetic components for CHD remain obscure [2], which makes it justifiable to identify novel CHD-causative genes.

## 2. Materials and Methods

### 2.1. Study Subjects

The present research project was fulfilled by the tenets of the Declaration of Helsinki. The local institutional ethics committee approved the protocols applied to this research. Written informed consent forms were signed by either the patients ≥18 years old or the guardians of the children <18 years old, before the start of the current investigation. For the current study, a pedigree suffering from autosomal-dominant CHD spanning four generations was enlisted from the Chinese Han-race population. A cohort of 384 unrelated volunteers with no CHD was enlisted as the control subjects from the same population in the same geographical area. All study subjects underwent clinical evaluation, including a review of personal, familial, and medical histories, physical examination as well as a transthoracic echocardiogram. Patients ranging from infants to adults were diagnosed with CHD by echocardiography, and some conditions were further validated surgically by a surgeon. Approximately 2 mL of blood samples were collected from every study individual. Extraction of genomic DNA was routinely conducted from study individuals’ blood leucocytes.

### 2.2. Genetic Assay

For a selected family member to construct a whole-exome library, a total amount of 2 μg of genomic DNA was utilized. The constructed whole-exome library was captured using the SureSelect Human All Exon V6 kit (Agilent Technologies, Santa Clara, CA, USA) and sequenced under the HiSeq 2000 platform (Illumina, San Diego, CA, USA), as per the manufacturer’s protocol. Bioinformatical assay of the datasets produced by whole-exome sequencing (WES, New York, NY, USA) was conducted as described elsewhere [81,82,83,84]. Sanger sequencing was conducted to validate the candidate variations discovered by WES and bioinformatical analysis. All coding exons along with splicing boundaries of the gene harboring a confirmed deleterious variation were sequenced in all the family members available and the 384 healthy subjects. Co-segregation analysis was carried out on the whole family of this investigation. Additionally, the Single Nucleotide Polymorphism database (dbSNP; https://www.ncbi.nlm.nih.gov/ accessed on 16 May 2020), the 1000 Genomes Projects (1000G; http://www.1000genomes.org/ accessed on 16 May 2020), and the Genome Aggregation Database (gnomAD; http://gnomad-sg.org/ accessed on 16 May 2020) were retrieved to verify its novelty.

### 2.3. Construction of Recombinant Plasmids

As described elsewhere [78], cDNA was prepared from the discarded human myocardium, which derived from a patient with tetralogy of Fallot who underwent radical surgery. The open reading frame of wild-type human *SOX18* (accession No. NM_018419.3) was produced by polymerase chain reaction (PCR) employing the Phusion^®^ DNA polymerase (NEB, Ipswich, MA, USA) and the primer pairs of 5′-GACGAATTCCGCGCTCCCGCGCTCCGTTC-3′ (forward) and 5′-GTCCTCGAGGAGGAAGCGCTGCAGGGACC-3′ (backward). The yielded full-length *SOX18* cDNA and the eukaryotic expression plasmid pcDNA3.1 were cut doubly by *Xho*I and *EcoR*I (NEB), respectively, extracted, and ligated to construct a recombinant wild-type SOX18-pcDNA3.1 plasmid. The sequence of SOX18 was confirmed to be wild-type by sequencing. The mutant-type SOX18-pcDNA3.1 plasmid containing the identified genetic variation was generated through site-targeted mutagenesis with the QuikChange Lightning Site-Directed Mutagenesis kit (Agilent) using the primer pairs of 5′-AACGCGGTGCTCAGCTAGATGCTGGGCAAAG-3′ (forward) and 5′-CTTTGCCCAGCATCTAGCTGAGCACCGCGTT-3′ (backward) and was checked by sequencing analysis. The NKX2.5-pEFSA plasmid expressing wild-type human NKX2.5 was described elsewhere [73,78]. A 1545 bp genomic DNA fragment (from −1554 to −10) of the human *NR2F2* gene (accession no. NC_000015.10) was PCR-amplified from human genomic DNA with the Phusion^®^ DNA polymerase (NEB) utilizing the primer pairs of 5′-GTGGCTAGCTGGTTGGTTTACCGGCATG-3′ (forward) and 5′-CACCTCGAGCCGCGATCAGCTCACTGAGC-3′ (backward), doubly cut using *Xho*I and *Nhe*I (NEB), and ligated to the pGL3-Basic vector with no promoter (Promega) to produce an *NR2F2* promoter-driven firefly luciferase expression plasmid (NR2F2-luc). Similarly, a 1979 bp fragment (from −4887 to −2909) of the human *GATA4* gene (accession No. NC_000008.11) was PCR-amplified from human genomic DNA with the Phusion^®^ DNA polymerase (NEB), and the primer pairs of 5′-GCGGCTAGCTGTGACTTCAAAAGTCTCT-3′ (forward) and 5′-CGCCTCGAGAGAATTTAACACTGTGAACG-3′ (backward), doubly digested with *Xho*I and *Nhe*I (NEB), and ligated to the pGL3-Basic vector (Promega), generating a *GATA4* promoter-driven firefly luciferase expression plasmid (GATA4-luc). All recombinant constructs were validated by sequencing analysis.

### 2.4. Cell Transfection and Reporter Assay

HeLa cells were seeded and cultivated as previously described [73]. The cells were maintained for 24 h to reach approximately 90% confluency and then transfected with the recombinant plasmids via the Lipofectamine 3000 Transfection Reagent (Invitrogen, Waltham, MA, USA), as previously described [73]. The internal control plasmid expressing renilla luciferase, pGL4.75 (Promega), was transfected for normalization of transfection efficiency. Unless otherwise indicated, 1.5 μg of NR2F2-luc or NGATA4-luc, 20 ng of pGL4.75, and 0.3 μg of each activating expression plasmid (wild-type SOX18-pcDNA3.1, Lys117*-mutant SOX18-pcDNA3.1 or NKX2.5-pEFSA, separately or in combination) were utilized. For each expression plasmid, at least three independent experiments were fulfilled in triplicate. The dual-luciferase activities were quantitatively measured as previously described [73], with a dual-luciferase reporter kit (Promega, Madison, WI, USA).

### 2.5. Statistics

The promoter activity was gauged using a ratio of firefly relative to renilla luciferase activity and shown as mean ± standard deviation as previously described [73]. The Student’s unpaired *t*-test was adopted to make a comparison between the two groups. When comparisons among multiple groups were performed, one-way ANOVA with a Tukey–Kramer HSD post hoc test was applied. A two-tailed *p*-value of <0.05 indicated a significant difference.

## 3. Results

### 3.1. Phenotypic Data of the Studied Family with CHD

**Figure 1:**
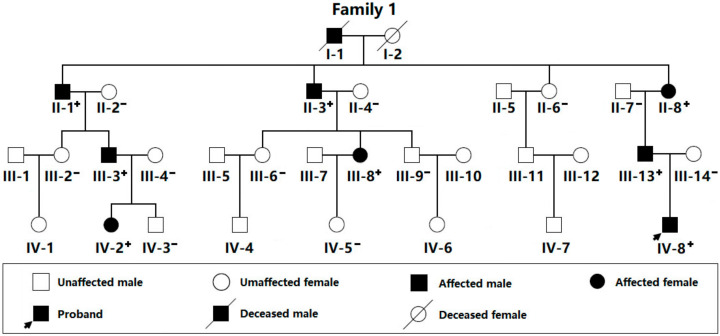
Pedigree affected by congenital cardiovascular abnormalities. A family member is identified by generation and number. “+” denotes a carrier of the identified *SOX18* variation in a heterogeneous status; “−” represents a non-carrier.

In the current research, shown in Figure 1, a 32-member family affected by CHD spanning four generations was identified from the Han-race population in China.

**Figure 2:**
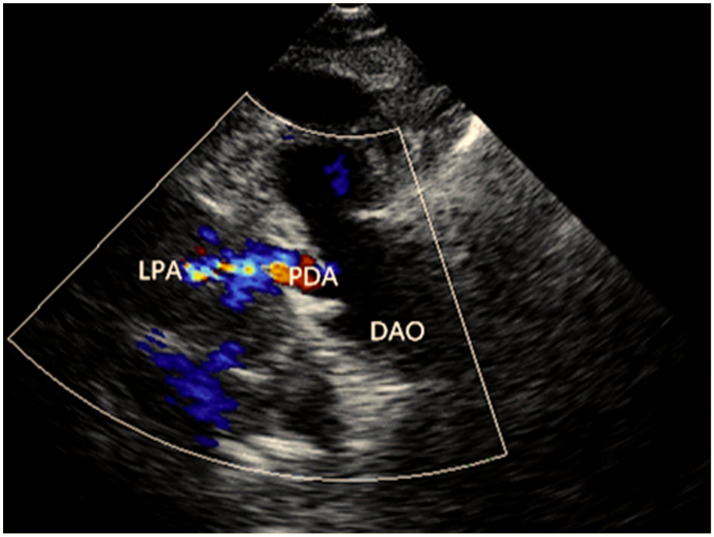
A representative echocardiographic image of the proband’s patent ductus arteriosus. The color flow image shows the presence of a patent ductus arteriosus between the left pulmonary artery and the descending aorta. DAO, descending aorta; LPA, left pulmonary artery; PDA, patent ductus arteriosus.

In this large family, 30 living family members were available, including 15 female family members and an equal number of male family members, with ages varying between 5 and 62 years. All nine affected family members were diagnosed with PDA by echocardiogram and underwent catheter-based interventional treatment for closure of PDA. A representative echocardiographic image of the proband’s PDA is given in Figure 2.

Additionally, there were four members (I-1, II-1, III-3, and IV-2 in Family 1) also suffering from PS. Genetic research of the family (Figure 1) unveiled that PDA was transmitted in the whole family with apparently autosomal-dominant inheritance, with complete penetrance. The index patient was a five-year-old boy, and his grandmother’s father (I-1 in Family 1) had been diagnosed with PDA and PS as well as lymphedema and died of chronic congestive cardiac failure at the age of 64 years. Furthermore, all CHD sufferers also had telangiectasia and hypotrichosis. No environmental factors prone to CHD were recognized among all family members. The clinical profile of the family members affected by CHD is presented in Table 1.

### 3.2. Discovery of a New CHD-Causative Variation in SOX18

WES was completed in six PDA-affected pedigree members (II-1, II-8, III-3, III-13, IV-2, and IV-8 in Family 1) as well as four healthy pedigree members (II-2, II-7, III-4, and III-14 in Family 1), which yielded ~21 Giga bases of sequence data per pedigree member, showing ~99% coverage of the human genome (GRCh37/hg19) with ~77% located to the target region. A mean of 17,028 nonsynonymous variations (varying from 15,410 to 18,136) per member passed filtering according to the likely transmission models, of which 12 nonsense and missense variations in heterozygous status passed filtering by ANNOVAR with a minor allele frequency of <0.1% and were possessed by the six PDA-affected members who underwent WES, as given in Table 2.

Sanger sequencing was performed for each variant, and only the *SOX18* variant c.349A>T (p.Lys117*) was co-segregated with the disease in the whole family. Of the other 11 genetic variations, 7 genetic variations (c.3037T>A in *ZNF644*, c.772T>A in *KLF7*, c.868C>G in *ASXL2*, c.4063T>C in *SYNE1*, c.1129A>T in *TGFBR1*, c.1147G>A in *EGR2* and c.1848A>T in *ZBTB1*) were also present in the healthy members, whilst the remaining 4 generic variations (c.566A>C in *KCNT2*, c.503G>A in *ZMAT3*, c.2812A>T in *ANK2* and c.1129A>T in *RBM20*) were absent in two affected members (II-3 and III-8) in the family. Hence, these 11 genetic variations were unlikely to be responsible for PDA in this family. The primers used to amplify the entire coding regions and splicing boundaries of the *SOX18* gene were shown in Table 3.

**Figure 3:**
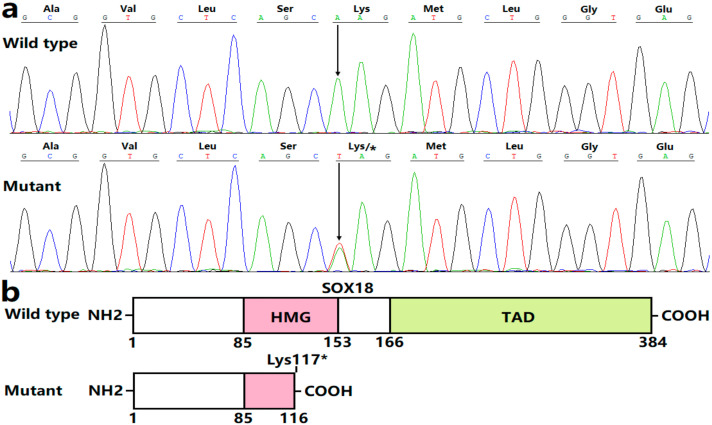
A novel *SOX18* variation accountable for congenital cardiovascular anomalies. (**a**) Sequence chromatograms displaying the heterogeneous SOX18 variation in the affected proband (mutant) in contrast to a homozygous wild type in a healthy individual (wild type). An arrow directs where the variation occurs. (**b**) Schemas describing the pivotal structural domains of SOX18. COOH: carboxyl-terminus; NH2: amino-terminus; TAD: transcriptional activation domain; HMG: high mobility group.

The chromatograms exhibiting the heterozygous *SOX18* variation (A/T) together with its wild-type homozygous bases (A/A) are given in Figure 3a. The schematic representations displaying the crucial structural motifs of both wild-type SOX18 and Lys117*-mutant SOX18 are drawn in Figure 3b. The detected *SOX18* variation, which was not found in 768 referential chromosomes, was not released in such databases as dbSNP, 1000G, and gnomAD, indicating a new *SOX18* variation. This variant in *SOX18*, NM_018419.3: c.349A>T; p.(Lys117*), was deposited in the Leiden Open Variation Database (https://databases.lovd.nl/shared/genes/SOX18; accessed on 9 May 2022), with a variant number of 0000848037.

### 3.3. Functional Failure of Lys117*-Mutant SOX18

**Figure 4:**
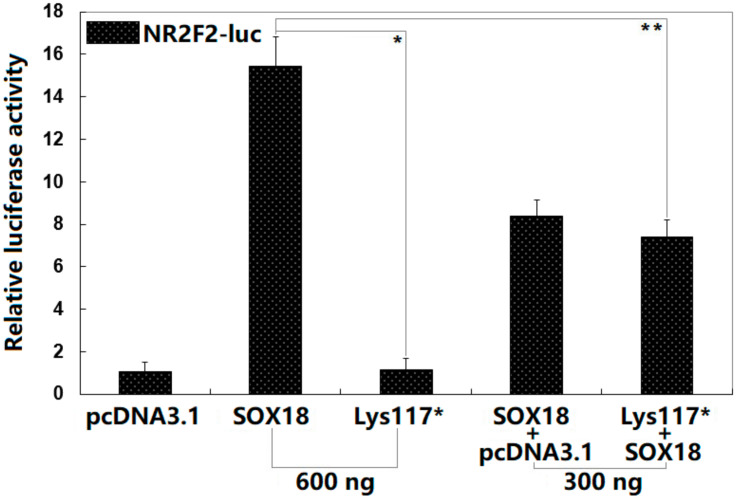
Functional impairment of SOX18 resulted from the variation. Reporter gene assay of the activation of the *NR2F2* promoter-driven luciferase in cultivated HeLa cells by wild-type SOX18 or Lys117*-mutant SOX18 (Lys117*), singly or together, revealed that the Lys117* variant had no transcription activity. For each recombinant expression plasmid, three independent experiments in vitro were fulfilled in triplicate. The Student’s *t*-test was employed to make the comparison between the two groups. Herein, * and ** denote *p* < 0.0001 and *p* < 0.001, respectively, in comparison with wild-type SOX18 (600 ng).

As illustrated in Figure 4, in cultured HeLa cells transfected with various expression plasmids, 600 ng of the empty pcDNA3.1 plasmid, 600 ng of the wild-type SOX18-pcDNA3.1 plasmid, 600 ng of the Lys117*-mutant SOX18 plasmid, 300 ng of the wild-type SOX18-pcDNA3.1 plasmid + 300 ng of the empty pcDNA3.1 plasmid, and 300 ng of the wild-type SOX18-pcDNA3.1 plasmid + 300 ng of the Lys117*-mutant SOX18 plasmid transactivated the *NR2F2* promoter by ~1-fold, ~15-fold, ~1-fold, ~8-fold, and ~7-fold, respectively. All the control and experimental values were compared with each other using one-way ANOVA followed by a Tukey–Kramer HSD post hoc test, with *p* = 8.132 × 10^−9^ (F = 145.51). Specifically, multiple comparisons were performed between pcDNA3.1 and SOX18 (*t* = 14.4067, *p* < 0.00001), pcDNA3.1 and Lys117* (*t* = 0.1233, *p* = 0.99974), pcDNA3.1 and pcDNA3.1 + SOX18 (*t* = 7.3733, *p* = 0.00001), pcDNA3.1 and pcDNA3.1 + Lys117* (*t* = 6.3733, *p* = 0.00003), SOX18 and Lys117* (*t* = 14.2833, *p* < 0.00001), SOX18 and pcDNA3.1 + SOX18 (*t* = 7.0333, *p* = 0.00001), SOX18 and SOX18 + Lys117* (*t* = 8.0333, *p* < 0.00001), Lys117* and pcDNA3.1 + SOX18 (*t* = 7.2500, *p* = 0.00001), Lys117* and SOX18 + Lys117* (*t* = 6.2500, *p* = 0.00003), and pcDNA3.1 + SOX18 and SOX18 + Lys117* (*t* = 1.0000, *p* = 0.62506). Here, the Lys117* + SOX18 group was used in vitro to mimic the pathogenic status of the patients carrying the heterozygous *SOX18* mutation; the SOX18 + pcDNA3.1 group was used to evaluate the potential dominant-negative effect of Lys117*-mutant SOX18 on wild-type SOX18, and the results revealed no dominant-negative effect of Lys117*-mutant SOX18 on wild-type SOX18.

### 3.4. Synergistic Activation between SOX18 and NKX2.5 Abrogated by the Lys117* Variation

**Figure 5:**
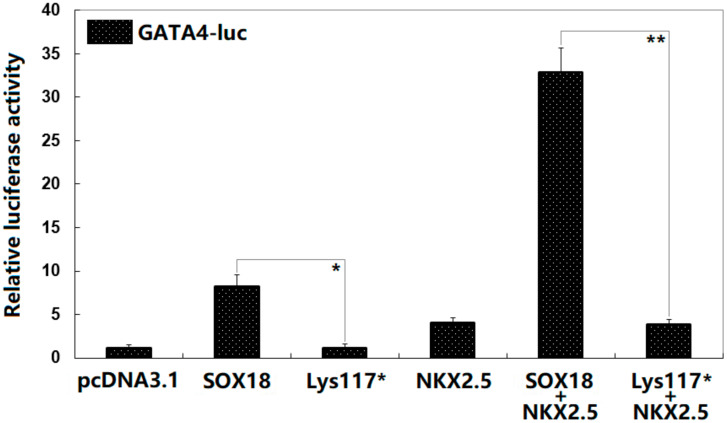
Synergistic activation between SOX18 and NKX2.5 abrogated by the variation. Dual-reporter gene measurement of the synergistical transactivation of the *NKX2.5*-promoter activity in cultured HeLa cells by SOX18 and NKX2.5 indicated that the synergy was abolished by the Lys117* variation. For each expression plasmid, three independent in vitro experiments were carried out in triplicate. A Student’s *t*-test was employed to compare two groups. Herein, * and ** mean * *p* < 0.001 and ** *p* < 0.0001, in contrast to the corresponding wild-type counterparts.

As exhibited in Figure 5, in cultured HeLa cells transfected with various expression plasmids, pcDNA3.1, SOX18, Lys117*, NKX2.5, SOX18 + NKX2.5, and Lys117* + NKX2.5 transactivated the *GATA4* promoter by ~1-fold, ~8-fold, ~1-fold, ~4-fold, ~33-fold, and ~4-fold, respectively. All the control and experimental values were compared with each other using one-way ANOVA followed by a Tukey–Kramer HSD post hoc test, with a *p* = 1.325 × 10^−11^ (F = 241.17). Specifically, multiple comparisons were performed between pcDNA3.1 and SOX18 (*t* = 7.1333, *p* = 0.00036), pcDNA3.1 and Lys117* (*t* = 0.0033, *p* = 1), pcDNA3.1 and NKX2.5 (*t* = 2.9333, *p* = 0.15919), pcDNA3.1 and SOX18 + NKX2.5 (*t* = 31.7000, *p* = 0), pcDNA3.1 and Lys117* + NKX2.5 (*t* = 2.7667, *p* = 0.19999), SOX18 and Lys117* (*t* = 7.1300, *p* = 0.00036), SOX18 and NKX2.5 (*t* = 4.200, *p* = 0.02428), SOX18 and SOX18 + NKX2.5 (*t* = 24.5667, *p* = 0), SOX18 and Lys117* + NKX2.5 (*t* = 4.3667, *p* = 0.01884), Lys117* and NKX2.5 (*t* = 2.9300, *p* = 0.15993), Lys117* and SOX18 + NKX2.5 (*t* = 31.6967, *p* = 0), Lys117* and Lys117* + NKX2.5 (*t* = 2.7633, *p* = 0.20089), NKX2.5 and SOX18 + NKX2.5 (*t* = 28.7667, *p* = 0), NKX2.5 and Lys117* + NKX2.5 (*t* = 0.1667, *p* = 0.99999), and SOX18 + NKX2.5 and Lys117* + NKX2.5 (*t* = 28.9333, *p* = 0).

**Figure 6:**
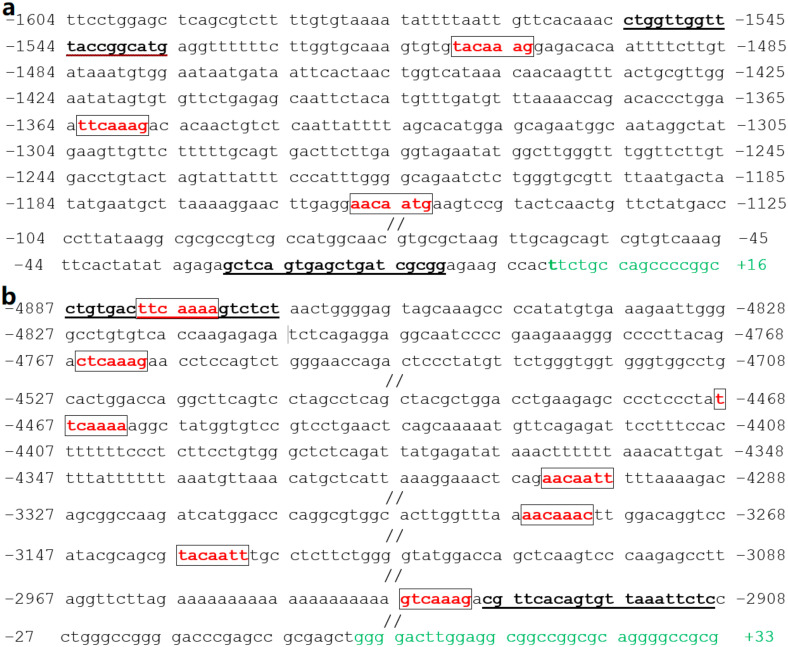
The SOX18-binding sites in the promoters of *NR2F2* and *GATA4*. (**a**) Multiple SOX18-binding sites mapped in the promoter of *NR2F2*. (**b**) Multiple variants of SOX18-binding sites mapped in the promoter of *GATA4*. Red color marks the SOX18-binding sites or their variants; green color: the first exon encoding mRNA; underlined bases: primer sequences.

Furthermore, for multiple SOX18-binding sites, the consensus sequences of the 5′-WWCAAWG-3′ (5′-A/TA/TCAAA/TG-3′) motifs in the promoter of *NR2F2* (accession no. NC_000015.10; transcript variant 1) were mapped and are shown in Figure 6a, while multiple SOX18-binding site variants in the promoter of *GATA4* (accession no. NC_000008.11; transcript variant 1) were mapped and are shown in Figure 6b. In addition to the SOX18-binding sites (highlighted by red color), the primer sequences (highlighted by bold underlines) and the first exons encoding mRNAs (highlighted by green color) are also shown, just in order to facilitate finding them from genomic DNA sequences.

## 4. Discussion

For the current investigation, a Chinese Han-race family affected by autosomal-dominant CHD spanning four generations was recruited. By WES and bioinformatics analysis of the pedigree members, a heterozygous *SOX18* variation, namely NM_018419.3: c.349A>T; p.(Lys117*), was discovered and substantiated via Sanger sequencing assay to co-segregate with the CHD phenotype in the entire family. This variation in *SOX18* was neither found in 768 referential chromosomes nor retrieved in dbSNP, 1000G, or gnomAD. Quantitative biological measurements unveiled that Lys117*-mutant SOX18 was unable to transcriptionally activate the promoters of *NR2F2* and *GATA4*, two CHD-causing genes [85]. Furthermore, the variation nullified the synergistic activation between SOX18 and NKX2.5, another gene responsible for CHD [85]. The findings strongly indicate that genetically compromised *SOX18* contributes to the molecular pathogenesis of CHD. However, although the *SOX18* mutation nicely segregates with the disease, and is a novel mutation, there is a view that an occurrence in more than one family is necessary to be able to call a gene disease-causing in CHD. Hence, it is necessary to perform a sequencing analysis of *SOX18* in other cohorts of PDA patients to identify more PDA-causative *SOX18* mutations in unrelated families with PDA.

*SOX18* was mapped on human chromosome 20q13.33, encoding a transcription factor with 384 amino acids, which belongs to a member of the SRY (sex-determining region Y)-related box (SOX) family of transcription factors [86,87]. Human SOX18 protein has two critical structural domains: the transcription activation domain (TAD) and the high-mobility group (HMG) domain. The N-terminal HMG box is essential for the sequence-specific binding of SOX18 to the consensus SOX DNA-binding motif of (A/T)(A/T)CAA(A/T)G (with the core DNA consensus sequence being AACAAT) in the promoters of its target genes, whilst the C-terminal TAD functions to transactivate target genes and also serves as a binding region for other transcriptional factors as transcriptionally cooperative partners of SOX18 [86,88]. SOX18 is abundantly expressed in the heart and vessels during embryogenesis, playing a critical role in embryogenic cardiovascular development and postnatal neovascularization, maybe by regulating the expression of target genes key to cardiovascular development, such as *NR2F2* and *GATA4*, alone or synergistically with its partners including NKX2.5 and MEF2C [73,78,86,87,88,89,90]. Moreover, loss-of-function variations in *NR2F2*, *GATA4,* and *NKX2.5* as well as *MEF2C* have been implicated in the occurrence of CHD [85,91,92]. In this investigation, the identified Lys117* variation was anticipated to create a truncated SOX18 protein lacking TAD and a part of the HMG domain, which was anticipated to exert a loss-of-function effect. Functional assays demonstrated that Lys117*-mutant SOX18 had no transactivation on its two representative downstream genes *NR2F2* and *GATA4*, alone or synergistically with its partner NKX2.5. Additionally, the present investigation unveiled that Lys117*-mutant SOX18 had no dominant-negative effect on wild-type SOX18. The findings suggest *SOX18* haploinsufficiency as an alternative genetic mechanism underpinning CHD that occurred in this family.

In vertebrates, at least 20 SOX genes have been cloned and subdivided into 10 groups (from group A to I), of which SOX18, SOX7, and SOX17 belong to group F of the SOX family (SoxF) [93]. It was demonstrated that three SOXF members are all co-expressed in the cardiovascular system and function to regulate cellular specification and tissue differentiation during cardiovascular development [93]. It may be attributable to abnormal cardiovascular morphogenesis that *SOX18* variation predisposes to CHD. In many species of animals, encompassing mice, zebrafish, Xenopus, and humans, SOX18 is expressed predominantly in the embryonic cardiovascular systems, playing a key role in cardiogenesis and vascular development, mainly via regulating the specification and differentiation of endothelial cells and cardiogenic mesoderm [86,87,93,94]. In Xenopus, injection of morpholinos against either *Sox18* or *Sox7* mRNAs led to partial inhibition of cardiogenesis, whereas co-injection of *Sox18* and *Sox7* morpholinos caused strong inhibition of cardiogenesis [94]. Furthermore, *Sox18* mRNAs rescued the effects of the *Sox7* morpholinos and vice versa, indicating that the two SOX proteins have functionally redundant roles [94]. In zebrafish, *Sox18* and *Sox7* morphants individually manifested minor vascular aberrations, whilst *Sox18*/*Sox7* double morphants displayed severe arterial-venous abnormalities as well as branching abnormalities of intersomitic vessels and loss of circulation in the trunk [86]. Additionally, only a part of *Sox7*^−/−^ zebrafish exhibited a lack of trunk circulation and a short circulatory loop, while the phenotypes were observed with complete penetrance in double *Sox18*^−/−^/*Sox7*^−/−^ zebrafish, suggesting that *Sox18* and *Sox7* exert a redundant role during cardiovascular morphogenesis [86]. In mice, aberration of heart looping, enlargement of the cardinal vein, and deformation of anterior dorsal aorta occurred in the *Sox17*-deficient embryos, while, in the *Sox17*/*Sox18* double-knockout embryos, more severe deformities occurred in the anterior dorsal aorta as well as head/cervical microvasculature, and an aberrant fusion of the endocardial tube as well as abnormal differentiation of endocardial cells was observed in some cases [93]. In the mice overexpressing SOX18 with a dominant-negative mutation, hemorrhages (rupture or distention of peripheral embryonic vessels), edema, and embryonic demise occurred due to cardiovascular defects [93]. By contrast, *Sox18*^−/−^ mice were viable and fertile, without apparent abnormality in their hearts and vasculature, suggestive of functional compensation by *Sox7* and *Sox17*, the two other SoxF genes [93]. In humans, loss-of-function alterations in both *SOX7* and *SOX17* have been related to CHD [73,78]. Moreover, mutations in *TFAP2B* have been reported to cause syndromic PDA by interfering with the inhibitory effect of TFAP2B on the canonical Wnt/β-catenin signaling pathway [95]. Given that all of the Xenopus, murine and human SOXF factors have a conserved β-catenin binding domain at the C-terminus and interact with β-catenin to repress the activity of β-catenin/TCF transcriptional complexes, and therefore suppress the Wnt/β-catenin signaling [86], the *SOX18* mutation identified in our study contributed to PDA probably by a similar mechanism. Taken together, these research results indicate that genetically compromised *SOX18*, one of three SOXF subgroup members that function redundantly, contributes to CHD in humans.

Previously deleterious mutations in multiple genes have been associated with syndromic PDA in humans, including *TBX1*-associated DiGeorge syndrome, *TBX5*-associated Holt–Oram syndrome, *PTPN11*-associated Noonan syndrome, *SMADIP1*-associated Mowat–Wilson syndrome, *CREBBP*-associated Rubinstein–Taybi syndrome, *TGFBR1/2*-associated Loeys–Dietz syndrome, *ABCC9*/*KCNJ8*-associated Cantu syndrome, and *TFAP2B*-associated Char syndrome [96]. Moreover, pathogenic mutations in several genes have been related to non-syndromic CHD with PDA being a prominent phenotype, including FLNA-related PDA and periventricular heterotopia and MYH11/ACTA2-related PDA and aortic aneurysm [96]. Additionally, there were common single nucleotide polymorphisms in several genes associated with enhanced susceptibility to non-syndromic PDA, including the rs987237 polymorphism in TFAP2β, the rs1056567 polymorphism in TRAF1, and the rs5186 polymorphism in AGTR1 [96]. In this investigation, SOX18 was identified as a new gene predisposing to non-syndromic PDA. However, whether SOX18 regulates the expression of these known PDA-related genes remains to be elucidated.

Previous investigations have unveiled that a premature translation termination codon may result in the degradation of mRNA in different types of organisms and cell lines through a mechanism named nonsense-mediated mRNA decay (NMD), a translation-dependent, multi-step process which monitors and degrades faulty or irregular mRNA [97]. In the present research, the nonsense mutation in *SOX18* created a premature translation termination codon; hence, the mutant *SOX18* mRNA was likely to undergo NMD, though not all nonsense mutations triggered it [98]. At present, we could not rule out NMD in the *SOX18* mutation carriers because of the unavailability of their cardiac tissue samples, where the mutant SOX18 protein might be expressed. Even though the mutant *SOX18* mRNA underwent NMD, the overall quantity of *SOX18* mRNA would be reduced by half, leading to haploinsufficiency, which was consistent with our functional results. Of note, downstream intron or pre-mRNA splicing, which brings about the deposition of a multi-protein complex, termed exon–junction complex, approximately 20–24 nucleotides upstream of each exon–exon junction, is necessary for the degradation of mRNA harboring a premature translation termination codon by the mechanism of NMD. Therefore, NMD could not occur in the context of cDNA constructs [97].

Notably, variations in the *SOX18* gene have previously been involved in hypotrichosis–lymphedema–telangiectasia syndrome as well as aortic dilation, pulmonary hypertension, dysmorphic face, renal failure, hydrocele, chylothorax, dysplastic nails, and cutis marmorata in humans [99]. In the present study, in addition to CHD, all the affected family members also manifested telangiectasia and hypotrichosis. Furthermore, the proband’s grandmother’s father also had lymphedema. Hence, these observational results expanded the phenotypic spectrum ascribed to *SOX18* variations.

## 5. Conclusions

The current research suggests *SOX18* as a new gene contributing to CHD, which is conducive to the clinical prognostic risk evaluation and timely prenatal prophylaxis of CHD in a subset of cases.

## Figures and Tables

**Table 1:** Clinical phenotypic data and *SOX18* variation status of the living pedigree members affected by congenital cardiovascular deformity.

Individual (Family 1)	Gender	Age (Years)	Cardiac Structural Deformities	SOX18 Variation (Lys117*)
II-1	Male	62	PDA, PS	+/−
II-3	Male	60	PDA	+/−
II-8	Female	55	PDA	+/−
III-3	Male	37	PDA, PS	+/−
III-8	Female	34	PDA	+/−
III-13	Male	31	PDA	+/−
IV-2	Female	13	PDA, PS	+/−
IV-8	Male	5	PDA	+/−

PDA, patent ductus arteriosus; PS, pulmonary stenosis; +/−, heterozygote for the *SOX18* variation.

**Table 2:** Nonsynonymous variations located in the candidate genes for congenital cardiovascular anomaly identified by whole-exome sequencing and bioinformatics analysis.

Chr	Position (GRCh37)	Ref	Alt	Gene	Variation
1	91,403,874	T	A	ZNF644	NM_201269.3: c.3037T>A; p.(Phe1013Ile)
1	196,434,495	A	C	KCNT2	NM_198503.5: c.566A>C; p.(Gln189Pro)
2	207,953,267	T	A	KLF7	NM_003709.4: c.772T>A; p.(Trp258Arg)
2	25,982,422	C	G	ASXL2	NM_018263.6: c.868C>G; p.(His290Asp)
3	178,745,488	G	A	ZMAT3	NM_022470.4: c.503G>A; p.(Gly168Glu)
4	114,239,688	A	T	ANK2	NM_001148.6: c.2812A>T; p.(Lys938*)
6	152,762,351	T	C	SYNE1	NM_182961.4: c.4063T>C; p.(Tyr1355His)
9	101,907,169	A	T	TGFBR1	NM_004612.4: c.1129A>T; p.(Arg377Trp)
10	112,590,865	T	G	RBM20	NM_001134363.3: c.3498T>G; p.(Cys1166Trp)
10	64,573,251	G	A	EGR2	NM_000399.5: c.1147G>A; p.(Asp383Asn)
14	64,990,070	A	T	ZBTB1	NM_001123329.2: c.1848A>T; p.(Leu616Phe)
20	62,680,521	A	T	SOX18	NM_018419.3: c.349A>T; p.(Lys117*)

Alt, alteration; Chr, chromosome; Ref, reference.

**Table 3:** Intronic primers to amplify the coding exons and splicing donors/acceptors of the *SOX18* gene.

Coding Exons	Forward Primers (5′→3′)	Backward Primers (5′→3′)	Amplicons (bp)
1 (a)	GGCCCTGAGCCGCTATATCT	CTTTGCCCACACCATGAAGG	457
1 (b)	CAGCTGGAATGCAGAGATCG	TCAGCTCCTTCCACGCTTTG	583
2 (a)	CAGCTGGAATGCAGAGATCG	CGGCCGGTACTTGTAGTTGG	672
2 (b)	AAGCGTGGAAGGAGCTGAAC	GGCTGCAGTTGAGGTACTGG	642
2 (c)	GCTCGCTGGCCTGTACTACG	TGTAACCCTGGCAACTCTGC	622

## Data Availability

The data supporting the discovery of the current research are available upon a reasonable request.
